# Translation Inhibition of the Developmental Cycle Protein HctA by the Small RNA IhtA Is Conserved across *Chlamydia*

**DOI:** 10.1371/journal.pone.0047439

**Published:** 2012-10-11

**Authors:** Jeremiah Tattersall, Geeta Vittal Rao, Justin Runac, Ted Hackstadt, Scott S. Grieshaber, Nicole A. Grieshaber

**Affiliations:** 1 Department of Oral Biology, College of Dentistry, University of Florida, Gainesville, Florida, United States of America; 2 Host-Parasite Interactions Section, Laboratory of Intracellular Parasites, National Institute of Allergy and Infectious Diseases, National Institutes of Health, Rocky Mountain Laboratories, Hamilton, Montana, United States of America; University of California Merced, United States of America

## Abstract

The developmental cycle of the obligate intracellular pathogen *Chlamydia trachomatis* serovar L2 is controlled in part by the small non-coding RNA (sRNA), IhtA. All *Chlamydia* alternate in a regulated fashion between the infectious elementary body (EB) and the replicative reticulate body (RB) which asynchronously re-differentiates back to the terminal EB form at the end of the cycle. The histone like protein HctA is central to RB:EB differentiation late in the cycle as it binds to and occludes the genome, thereby repressing transcription and translation. The sRNA IhtA is a critical component of this regulatory loop as it represses translation of *hctA* until late in infection at which point IhtA transcription decreases, allowing HctA expression to occur and RB to EB differentiation to proceed. It has been reported that IhtA is expressed during infection by the human pathogens *C. trachomatis* serovars L2, D and L2b and *C. pneumoniae*. We show in this work that IhtA is also expressed by the animal pathogens *C. caviae* and *C. muridarum*. Expression of HctA in *E. coli* is lethal and co-expression of IhtA relieves this phenotype. To determine if regulation of HctA by IhtA is a conserved mechanism across pathogenic chlamydial species, we cloned *hctA* and *ihtA* from *C. trachomatis* serovar D, *C. muridarum, C. caviae* and *C. pneumoniae* and assayed for rescue of growth repression in *E. coli* co-expression studies. In each case, co-expression of *ihtA* with the cognate *hctA* resulted in relief of growth repression. In addition, expression of each chlamydial species IhtA rescued the lethal phenotype of *C. trachomatis* serovar L2 HctA expression. As biolayer interferometry studies indicate that IhtA interacts directly with *hctA* message for all species tested, we predict that conserved sequences of IhtA are necessary for function and/or binding.

## Introduction

*Chlamydiaceae* are gram negative, obligate intracellular bacterial pathogens, with different species causing a wide range of diseases in both humans and animals. *Chlamydia trachomatis* biovars are major pathogens in humans and infect the urogenital tract and the eye in a serovar dependent manner. The urogenital serovars of *C. trachomatis* are the leading cause of bacterial sexually transmitted disease (STD) worldwide, the complications of which can lead to serious sequelae including pelvic inflammatory disease, ectopic pregnancies and infertility [1], [2]. The ocular serovars of *C. trachomatis* cause trachoma, a chronic follicular conjunctivitis that results in extensive scarring and blindness and are considered the leading cause of infectious preventable blindness in developing countries [3]. *C. pneumoniae* is the causative agent of human respiratory illnesses and is responsible for approximately 10% of community acquired pneumonia and 5% of bronchitis and sinusitis cases [4]. *Chlamydia* which cause pathology in animals include *C. abortus* (abortion and fetal loss in ruminants), *C. felis* (conjunctivitis and respiratory problems endemic in cats), *C. caviae* (conjunctivitis in guinea pigs) and *C. psittaci* (affects conjunctiva, respiratory system and gastrointestinal tract of birds) and can lead to zoonotic infections in humans. *C. muridarum* infects members of the family Muridae and is often used as a genital infectious model of *C. trachomatis* genital disease [5].

The chlamydial developmental cycle occurs entirely within a membrane bound parasitophorous vesicle termed an inclusion. Once internalized, *Chlamydia* undergo dramatic physiological and morphological changes alternating between two distinct forms, the elementary body (EB) and the reticulate body (RB). The metabolically inert EB is the infectious unit, able to survive extracellularly and disseminate to invade susceptible host cells. Upon infection of a host cell the EB differentiates to the non-infectious, metabolically active RB which divides repeatedly by binary fission. Late in the infection, a subset of RBs differentiate into the terminal but infectious EB form while the remaining RBs continue to replicate, resulting in asynchrony of the chlamydial developmental cycle [6]. The terminally differentiated EBs infect neighboring cells upon EB release due to cell lysis or inclusion extrusion [7].

It is not yet clear as to how differentiation between the EB and RB cell forms is regulated. Two proteins central to differentiation are HctA and HctB, both lysine rich, highly basic proteins with primary sequence homology to the eukaryote histone H1 [8]–[11]. Both proteins are expressed late in development, concomitant with RB to EB conversion and repress transcription and translation by binding to and occluding the genome [8], [9], [11]–[14]. Upon infection, the characteristic core of condensed chromatin of the EB is dispersed as differentiation into the pleomorphic RB occurs. Although nucleoid dispersion and gene transcription occurs within the first few hours of infection, HctA and HctB levels remain fairly constant indicating that these two proteins are no longer functioning to condense the genome in early chlamydial developmental forms [14]–[16]. A metabolite produced by the non-mevalonate methylerythritol phosphate (MEP) pathway of isoprenoid synthesis, thought to be 2-C-methyl-D-erythritol 2,4- cyclodiphosphate (MEC), was found to disrupt the chromosomal interactions of both HctA and HctB. It is hypothesized that MEC is a general modulator of EB germination [14], [16].

Expression of HctA is very tightly regulated and is repressed by the small non-coding RNA (sRNA), IhtA until RB to EB re-differentiation [17]. Bacterial sRNAs regulate the translation or stability of a target messenger RNA during specific developmental stages or stress conditions (reviewed in [18]–[21]. IhtA is transcribed early in infection and represses the translation of *hctA* mRNA without affecting the stability of the mRNA. Late in infection, IhtA transcription decreases allowing HctA synthesis to occur and RB to EB differentiation to proceed. In this study we demonstrate that the regulation of HctA by the sRNA IhtA is conserved in the important chlamydial pathogens of both humans and animals.

## Results

### The *ihtA* Gene Loci is Present and Expressed in Diverse Chlamydial Species

**Figure 1:**
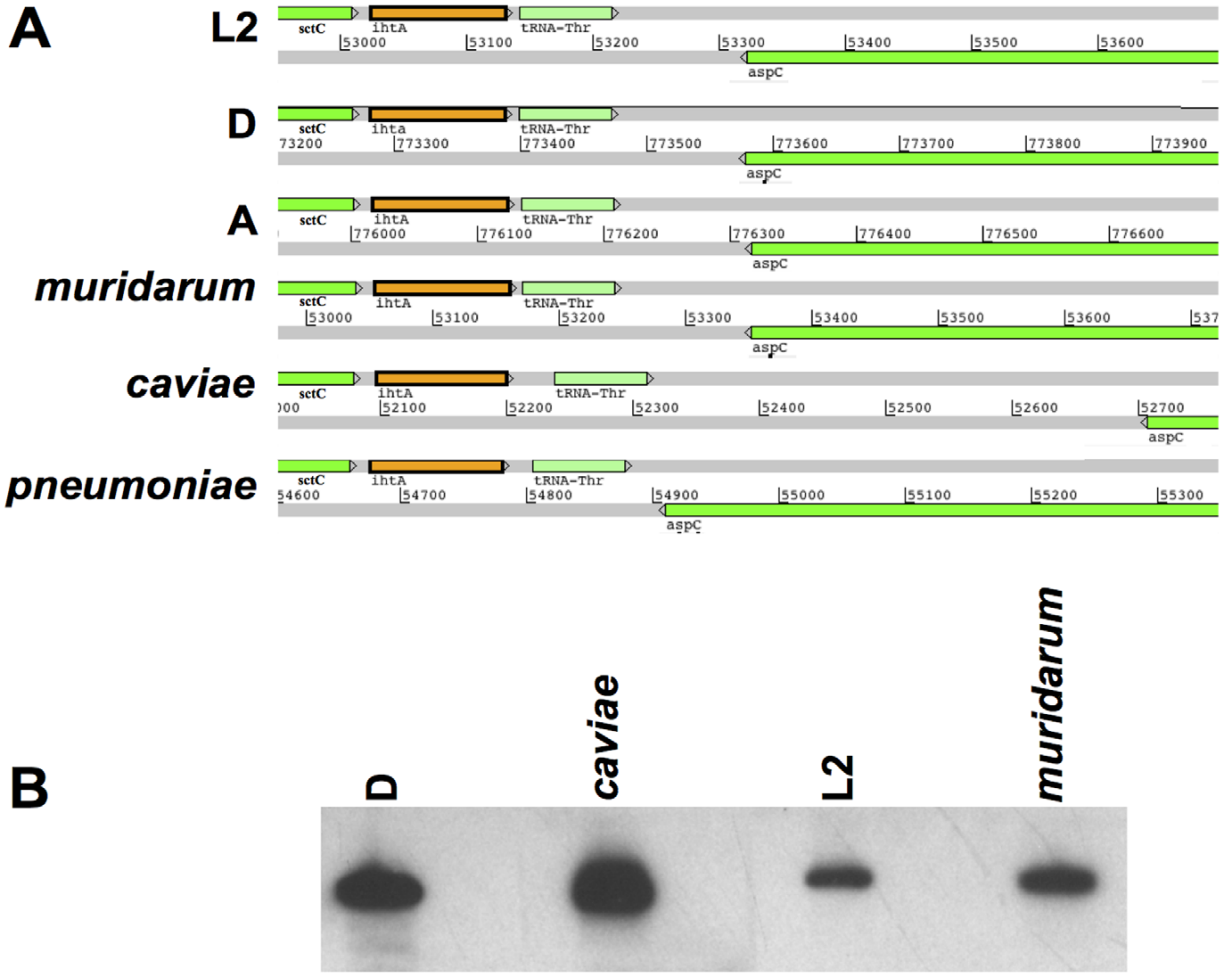
IhtA is expressed in diverse chlamydial species. (A) The IhtA containing genetic loci of *C. trachomatis* serovar L2, D and A and and the more disparate *C. muridarum*, *C. caviae* and *C. pneumoniae* were obtained and aligned by searching the complete genomes of the respective bacteria. (B) Northern analysis of IhtA expression during infection with *C. trachomatis* serovar D, *C. caviae*, *C. trachomatis* serovar L2 and *C. muridarum*. HeLa cell cultures were infected with *C. trachomatis* serovar L2, *C. caviae*, and *C. muridarum* for 24 h while *C. trachomatis* serovar D was grown for 48 h in 1 well of a 6 well plate prior to harvesting of sRNA. The entire sRNA sample was separated on a 10% TBE-Urea gel and probed with a species specific biotinylated PCR fragment.

The gene encoding *ihtA* is located on the positive strand in the IGS between the type III secretion system outer membrane ring protein, *sctC* and tRNA-Thr of *C. trachomatis* serovar L2 434. The promoter for *ihtA* is actually embedded in the 3′ end of the *sctC* open reading frame (ORF) [17]. The gene *aspC* is encoded just downstream of tRNA-Thr on the negative strand. This same genomic organization holds true for all sequenced *Chlamydiaceae* including the *C. trachomatis* serovars D (genital specific) and A (ocular specific), as well as the human respiratory pathogen *C. pneumoniae,* the Muridae species *C. muridarum* and the guinea pig specific *C. caviae* (Fig. 1A). It has recently been shown that the IhtA transcript is expressed by *C. pneumoniae, C. trachomatis* serovar D and *C. trachomatis* serovar L2b/UCH-1/proctititis during the course of infection [22]–[25]. In order to determine if sRNA control of HctA expression is conserved across pathogenic *Chlamydia* we first sought to confirm the expression of IhtA in *C. muridarum* and *C. caviae*. We performed Northern analysis of sRNA samples isolated from host cells infected with *C. muridarum* and *C. caviae* using RNA isolated from *C. trachomatis* serovar D and serovar L2 infection as controls. Expression of IhtA could be detected in all cases (Fig. 1B).

**Figure 2:**
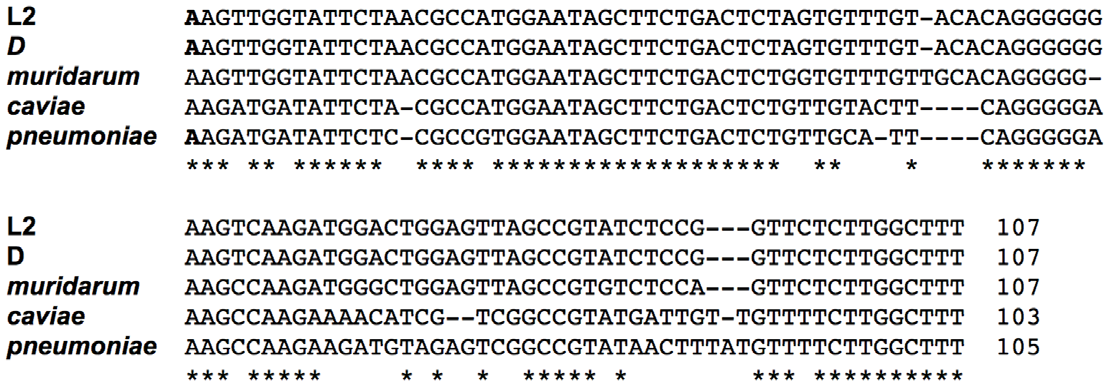
IhtA sequence is conserved across species. The sequence of *ihtA* of *C. trachomatis* serovar D, *C. muridarum, C. caviae*, and *C. pneumoniae* compared to *C. trachomatis* serovar L2. The TSS of serovars L2 and D and *C. pneumoniae* ihtA (indicated in bold) has been experimentally proven while the TSS of the other *Chlamydia* is predicted.

**Figure 3:**
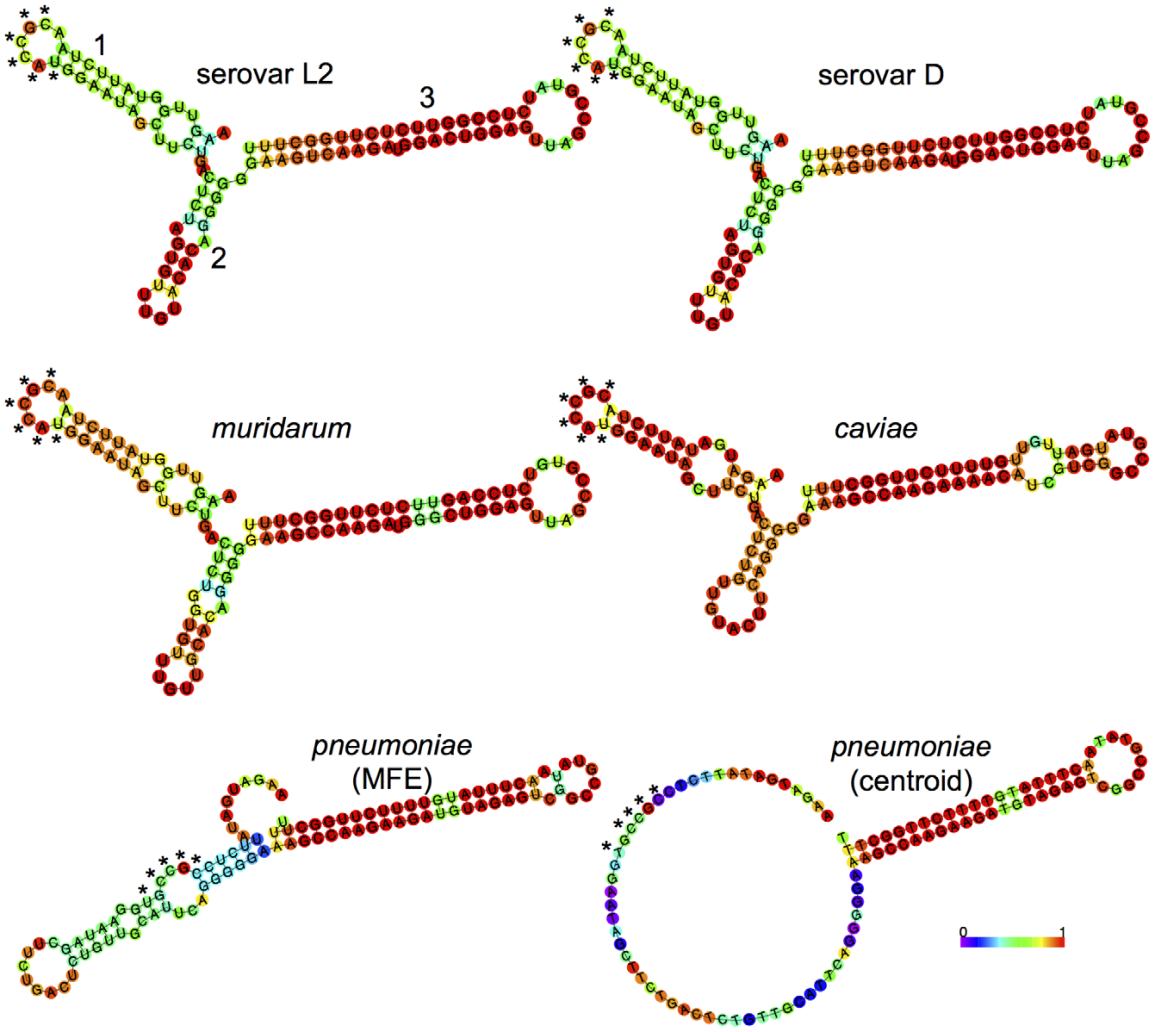
Structural prediction of species IhtA. Structural predictions were made using the RNAfold server contained in the Vienna websuit (http://rna.tbi.univie.ac.at). The three stem:loops of L2 IhtA are indicted numerically. Only the minimal free energy (MFE) of *C. trachomatis* serovars L2 and D, *C. muridarum* and *C. caviae* are displayed as the centroid structures are identical to the MFE structures indicating high confidence in the predictions. Both the MFE and centroid structures of *C. pneumoniae* are shown as they differ significantly. The * indicates the region of IhtA complimentary to the first 6 nucleotides of *hctA.* Base pair probabilities calculated by the web server are color coded 0–1, with higher numbers corresponding to higher confidence.

We had previously identified the transcription start site (TSS) to be a residue located 8 nt downstream of the beginning of the IGS by primer extension analysis [17]. Albrecht *et a*l, however identified the TSS to be the A residues 6 nt downstream of the previously identified TSS using a deep sequencing approach [24]. This TSS was confirmed in Serovar D by AbdelRahman et al by 5′ RACE [22], by our lab in serovar L2 (data not shown) and in *C. pneumoniae* [25]. Using this consensus TSS and the 3′ end identified by AbdelRahman et al in serovar D and our lab in serovar L2 (unpublished data), we predicted the sequence of the IhtA transcript in *C. muridarum* (105 nt), *C. caviae* (103 nt) and *C. pneumoniae* (105 nt). When aligned, the different species *ihtA* displayed a high level of identity to the *ihtA* of *C. trachomatis* serovar L2, with serovar D and *C. muridarum* at the highest (100% and 96% respectively) and *C. caviae* and *C. pneumoniae* at the lowest (70% and 69% respectively)(Fig. 2). Using the RNAfold web server of the Vienna RNA Websuit [26], we predicted the secondary structure of all five species IhtAs to determine if secondary structure was similar. RNAfold predicts both the minimum free energy (MFE) [27] and centroid [28] secondary structures of RNA molecules. The more similar the MFE and centroid structures, the more reliable the prediction [26]. The predicted structures of *C. trachomatis* serovars L2 and D, *C. muridarum* and *C. caviae* IhtA were quite similar, with each structure containing three stem:loops (Fig. 3). As the MFE and centroid structures of each species were identical, only the MFE structure is shown in Fig. 3. The open loops, which in general are the structures free for initial interaction with the sRNA target, were highly conserved among these four species. Interestingly, the loop of stem:loop 1 contains a 6 nt region which is complimentary to to the first 6 nt of the *hctA* ORF (denoted with an asterisks in Fig. 3). In contrast, the MFE and centroid structural predictions of *C. pneumoniae* IhtA displayed little similarity indicating a lack of reliability in the predictions (Fig. 3). The centroid structure was almost completely open with the exception of stem:loop 3 which is likely the terminator. However, the region complimentary to the first 6 nt of *hctA* is partially open in the MFE structure and completely open in the centroid structure which could allow for target interaction.

### IhtA Binds Directly to the *hctA* RNA Message

**Figure 4:**
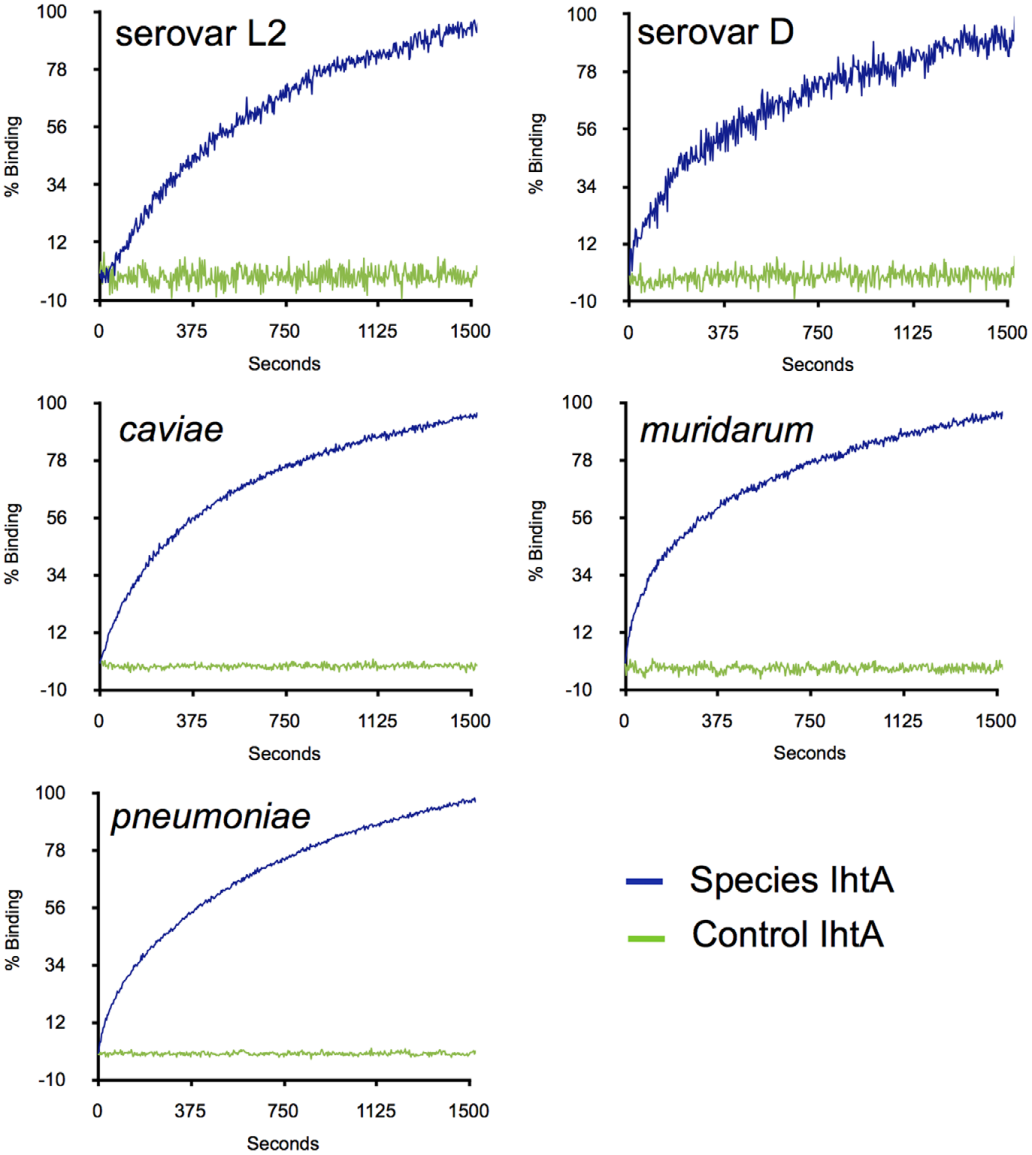
IhtA of each species interacts with the cognate *hctA* target mRNA in vitro. Run off *hctA* transcripts made from species specific PCR fragments were annealed to biotinylated oligo T and bound to BLI sensor tips. *hctA* bound tips were incubated with species specific native IhtA or antisense serovar L2 (scrambled control) IhtA and the change in reflected light indicating RNA:RNA binding was measured over time.

In general sRNA regulatory molecules modulate gene expression via direct base pairing with their target mRNA and more rarely, by altering the activity of a protein which in turn impacts gene regulation [29]. As it is unlikely that *E. coli* produces a protein specific for IhtA regulation we hypothesize that IhtA represses *hctA* translation by interacting directly with the *hctA* message. To determine if the IhtAs of all five species could interact with their cognate *hctA* mRNA, we measured IhtA to *hctA* binding in real time using biolayer interferometry (BLI). Briefly, *hctA* run off transcripts of each species were annealed to a biotinylated DNA oligo and bound to a streptavidin coated optical sensor tip. The sensor was then dipped into a solution containing species specific IhtA run off transcripts and RNA:RNA binding was determined in real time by measuring the change in reflected light through the sensor tip. Antisense serovar L2 IhtA was used as a scrambled non-binding control in each case. The data was normalized to percent maximum change in reflected light over time and compared to scrambled transcript (Fig. 4). These measurements indicate that IhtA of each species is capable of interaction with its cognate *hctA* target mRNA in vitro.

### IhtA Functions to Repress HctA Expression in Diverse Chlamydial Species

**Figure 5:**
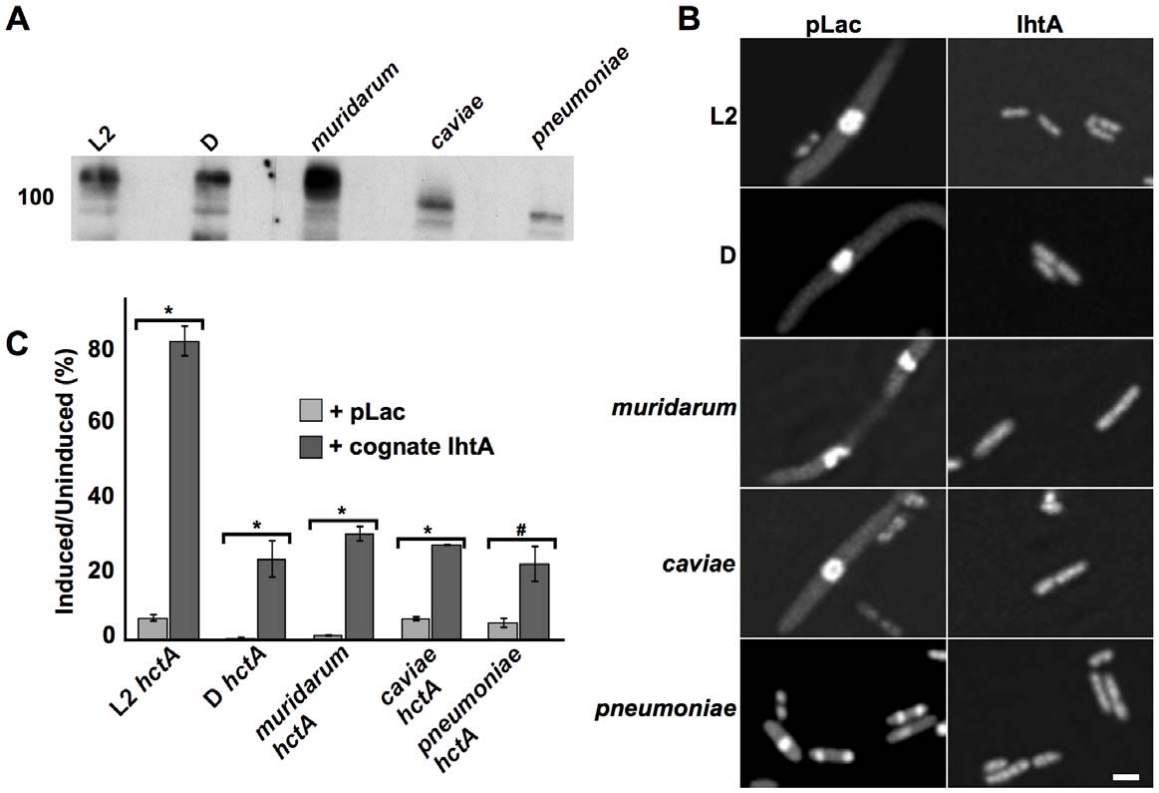
Repression of HctA translation by IhtA is a conserved mechanism. (A) Northern analysis of expression of species IhtA in *E. coli*. The *ihtA* gene, including 5′UTR was PCR amplified from the genomes of the indicated chlamydial species, cloned into pLac and expressed in DH5alphaPRO *E. coli.* sRNA was isolated from overnight cultures using the *mir*Vana miRNA Isolation kit (Ambion, Inc.). Northern analysis was performed on 2 µg of each sample separated on a 10% TBE-urea acrylamide gel which was transferred to BrightStar-Plus Nylon membrane and probed with a species specific biotinylated *ihtA* PCR fragment. (B) DRAQ5 DNA staining of *E. coli* induced to express the indicated species specific HctA and either empty vector (pLac) or the cognate IhtA. Condensed DNA appears as a central brightly fluorescent sphere (bar equals 2.5 um). C) Representative viability assay of *E. coli* expressing either species specific *hctA* alone (+pLac, light grey bars) or species specific *hctA* and IhtA (+cognate *ihtA*, dark grey bars). Each condition was performed in triplicate with a minimum of three repeats. The bars represent the S.E.M of each triplicate. The * indicates p value <0.001 and # indicates p value = 0.003.

Translation repression of serovar L2 *hctA* by the sRNA IhtA can be monitored by assaying for relief of both nucleoid condensation and the repressive growth phenotype induced by HctA over-expression in *E. coli* [9], [17]. Therefor, to determine if sRNA regulation of *hctA* translation is conserved across *Chlamydiaceae* we first PCR amplified *ihtA* from *C. trachomatis* serovar D, *C. muridarum, C. caviae* and *C. pneumoniae* genomic DNA and cloned the resulting fragment into pLac using the primers indicated in Table S1. We have shown previously that IhtA is constitutively expressed in *E. coli* when the promoter region is included, therefor all *ihtA* clones included 5′UTR [16], [17]. Northern analysis of sRNA isolated from overnight cultures indicate that the IhtA transcript of each species tested was constitutively expressed (Fig. 5A). The coding sequence of IhtA’s target, *hctA* was PCR amplified from *C. trachomatis* serovar D, *C. muridarum*, *C. caviae* and *C. pneumoniae* genomic DNA and subcloned into the pTet vector under the control of the *tet* promoter. Ectopic expression of each species of HctA resulted in a dramatic condensation of the *E. coli* nucleoid as monitored by DRAQ5 staining (Fig. 5B). Co-expression of the species *hctA* with the cognate IhtA relieved this phenotype indicating that each species IhtA could suppress the translation of its cognate *hctA*. In addition, over-expression of each species HctA resulted in repression of growth (Fig. 5C, light grey bars). In each case this growth repression was relieved to a significant level by co-expression with the cognate IhtA with p values <0.001 in the case of *C. trachomati*s serovars L2 and D, *C. muridarum* and *C. caviae* and a p value = 0.003 for *C. pneumoniae* (Fig. 5C, dark grey bars). Although the levels of rescue of *C. trachomati*s serovar D, *C. muridarum*, *C. caviae* and *C. pneumoniae* do not approach that of *C. trachomatis* serovar L2, these data taken together suggest a conservation of IhtA function.

### Conserved Regions of *ihtA* are Important to Function

**Figure 6:**
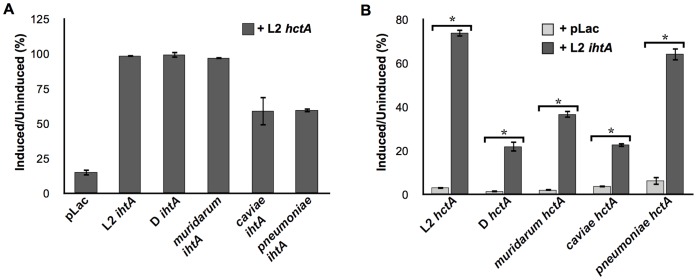
Serovar L2 HctA expression is repressed by species IhtA. A) Growth viability of *E. coli* co-expressing serovar L2 HctA and *C. trachomatis* serovar D, *C. muridarum, C. caviae*, or *C. pneumoniae* IhtA. *E. coli* co-expressing *hctA* and empty vector (pLac) or L2 IhtA served as negative and positive controls respectively. Each condition was performed in triplicate with a minimum of three repeats. The bars represent the S.E.M of each triplicate. B) Growth viability of *E. coli* co-expressing species *hctA* and *C. trachomatis* serovar L2 IhtA. Each condition was performed in triplicate with a minimum of three repeats. The bars represent the S.E.M of each triplicate. The * indicates a p value ≤0.001.

As indicated in Figures 2, *ihtA* sequence is quite similar across *Chlamydia*. We therefor sought to determine if IhtA from *C. trachomatis* serovar D, *C. muridarum*, *C. caviae* and *C. pneumoniae* could functionally substitute for that of serovar L2 IhtA. To this end, *E. coli* were co-transformed with *C. trachomatis* serovar L2 *hctA* and species specific *ihtA* and monitored for growth (Fig. 6A). Expression of IhtA isolated from *C. trachomatis* serovars D and *C. muridarum* rescued the serovar L2 HctA over-expression growth defect to levels similar to serovar L2 IhtA controls. Co-expression of *C. caviae* and *C. pneumoniae* IhtA with *C. trachomati*s serovar L2 *hctA* resulted in an intermediate rescue (average of 60% rescue over three separate experiments). Although the IhtA sRNAs from the more distantly related *Chlamydia* did not rescue growth repression to the same levels as that of L2 IhtA, the L2 HctA growth phenotype was significantly relieved in all cases.

The converse experiment in which serovar L2 IhtA was co-expressed with *C. trachomatis* serovar D, *C. muridarum*, *C. caviae* and *C. pneumoniae hctA* also resulted in relief of HctA induced growth repression (Fig. 6B). Although the growth phenotype was significantly rescued in each case (p values ≤0.001), the rescue was more variable as was the case for IhtA co-expression with the cognate *hctA*. Nevertheless, that IhtA is relatively interchangeable indicates that the overall function of IhtA in *hctA* translation repression is conserved. Additionally, these data suggest that the sequences and/or structural features conserved between species may be important for functional activity.

## Discussion

A defining characteristic of the *Chlamydiaceae* family is the biphasic developmental cycle. All bacteria in this family share this basic life cycle consisting of specialized cell types for cell binding and entry (EB) and intracellular replication (RB). Differentiation between the two cell types is in part controlled by the expression of the histone-like proteins HctA and HctB. We previously reported the identification of a small non-coding RNA, termed IhtA which acts as a regulatory molecule controlling the expression of the HctA protein at the RB to EB transition point in *C. trachomatis* serovar L2 [17]. Here we show that *ihtA* is conserved across all vertebrate pathogenic *Chlamydia*. *IhtA* is contained in the intergenic region of the chromosome between *sctC* and the thr-tRNA in each of these organisms. Although regulation of cell type differentiation is poorly understood, it is appreciated that the expression of HctA is a critical component of the cascade leading to EB differentiation. As the correct timing of HctA expression is critical to the infectious cycle, it could be predicted that exquisite control of *hctA* translation by IhtA would be a conserved mechanism. Indeed, micro-array analysis and RNA sequencing of RNA isolated from a selection of human chlamydial pathogens demonstrate that IhtA is expressed upon infection of a host cell [22]–[25] and Fig. 1). In addition, it has been shown that the expression pattern of IhtA in *C. trachomatis* serovar D and *C. pneumoniae* is similar to that of serovar L2, both over a time course of infection and during the RB to EB differentiation process [22], [23].

Ectopic expression of *hctA* cloned from *C. trachomatis* serovar D, *C. muridarum*, *C. caviae*, and *C. pneumoniae* in *E. coli* resulted in the characteristic condensed nucleoid and growth repression observed in *E. coli* expressing serovar L2 HctA. Co-expression of IhtA cloned from these different species relieved both phenotypes, presumably via repression of HctA translation. It is curious that although relief of the growth phenotype of species HctA overexpression by the co-expression of the cognate IhtA was significant (Fig. 4), rescue was not to the same extent as that of the serovar L2 IhtA:*hctA* partnership. This variable extent of rescue of the species HctA was again evident with serovar L2 interspecies rescue. As *E. coli* is used as a surrogate system it is difficult to interpret these nuances but several possibilities exist. The HctA protein has a bimodal sequence conservation. The majority of the conserved amino acid sequence in HctA is located in the N-terminal domain of the protein. The first 10 amino acids of HctA of all five species tested are 100% conserved. Outside of this region (the remaining +/−116 aa depending on species) identity between *C. trachomatis* serovar L2 and *C. trachomatis serovar D*, *C. muridarum, C. caviae and C. pneumoniae* falls to 98%, 98%, 84% and 82% respectively. Interestingly, the more divergent C-terminal region contains the DNA binding domain of HctA [30]. This suggests there may be significant differences in the way these proteins interact with *E. coli* DNA, potentially contributing to the different levels of rescue observed.

Although not directly tested it is possible that HctA did not express at consistent levels across species and/or experiments. This seems less likely as all constructs are expressed from the same promoter although we did not account for differential codon usage when expressed in *E. coli.* Variability in expression across species is certainly true for IhtA (which is expressed from its native promoter in our system) as evidenced by Northern analysis. We show that each species IhtA functionally substituted for serovar L2 IhtA and effectively repressed serovar L2 HctA expression in *E. coli*. The more distantly related *C. caviae* and *C. pneumoniae* did not rescue to wild type levels. As the molar ratio of IhtA:*hctA* required for full repression of translation, either in the *E. coli* surrogate system or in vivo where there may be competing targets, is unknown. Therefor as *C. caviae* and *C. pneumoniae* IhtA appear not to express as well as *C. trachomatis* serovars L2 and D and *C. muridarum* in *E. coli*, it is difficult to predict if partial rescue of serovar L2 HctA over-expression is due to functionality or dosage. Nevertheless, as IhtA is expressed and regulated in vivo in all chlamydial species tested [14], [17], [22]–[25] and IhtA relieved growth repression when co-expressed with *hctA* in *E.coli* (this manuscript), a conservation of function is suggested.

IhtA is a trans-encoded sRNA, present at a genetic location distinct from its target [17]. Trans-encoded sRNAs bind their target mRNAs via short interrupted base pairings which may contain internal bulges and include non-canonical base pairing, thus interacting sequences are often difficult to predict [31]–[33] (reviewed in [29]). The evidence to date suggests that IhtA functions by binding directly to *hctA* and not through a protein intermediate, however this proposal has not been directly tested. Using biolayer interferometry we show in real time that IhtA from all species tested are capable of interacting directly to its target mRNA, *hctA*.

As interaction between two RNA molecules is mediated in most cases by Watson-Crick base pairing it is likely that the interaction between IhtA and the *hctA* mRNA occurs through base pairing of conserved residues in both molecules. That IhtA from each species is able to repress serovar L2 *hctA* translation supports this prediction. The first 31 nucleotides of the *hctA* ORF of all five species tested are 100% conserved. Over the remaining +/−347 nt identity between *C. trachomatis* serovar L2 and serovar D, *C. muridarum, C. caviae* and *C. pneumoniae* falls to 99%, 85%, 74% and 77% respectively. IhtA of each of the five species is highly conserved on a sequence level and the predicted structures of IhtA of four of the five species tested are similar. As noted in the results section, the structure of *C. pneumoniae* was difficult to predict and the structures predicted are of low confidence. However, in each case, including *C. pneumoniae*, a 6 nt stretch of IhtA which is complimentary to the first 6 nt of the *hctA* ORF beginning with and including the ATG start site, resides in what is predicted to be an unpaired open region of IhtA. As these structures are not experimentally determined it is perhaps premature to extrapolate to biological function, however it is appealing to predict that this region is important for direct RNA:RNA interactions leading to inhibition of *hctA* message translation by occluding the start site.

As IhtA and HctA expression is similarly regulated across species during infection, species IhtA directly binds to the cognate *hctA* in vitro and IhtA co-expressed with *hctA* in various combinations rescues growth repression to a significant degree, we suggest that the IhtA/*hctA* interaction is an important conserved regulatory circuit and part of the RB to EB differentiation program shared by chlamydial pathogens.

## Materials and Methods

### Chlamydia Growth and Cell Culture

*C. trachomatis* serovar L2 (strain LGV 434), *C. trachomatis* serovar D (strain UW-3/Cx), *C. pneumoniae* (strain AR-39), *C. muridarum* and *C. caviae* were propagated in HeLa 229 cells and isolated by Renografin (Squibb) density gradient centrifugation as described previously [34]–[36].

### RNA Isolation and Northerns

sRNA was isolated from both bacterial cultures and infected HeLa monolayers using the *mir*Vana miRNA Isolation kit as described by the manufacturer (Ambion, Inc.). *E. coli* expressing IhtA were pelleted and washed twice in ice cold PBS prior to sRNA isolation. sRNA was purified from HeLa 229 cultures infected with *C. trachomatis* serovar L2 LGV 434, *C. muridarum* or *C. caviae* at 24 h PI and *C. trachomatis* serovar D at 48 h PI. Northern analysis was performed on sRNAs separated on a 10% TBE-urea acrylamide gel and transferred to BrightStar-Plus Nylon membrane (Ambion, Inc.). Membranes were hybridized overnight with the appropriate biotinylated probe at 42°C in ULTRAhyb (Ambion, Inc.). Nonisotopic IhtA probes were generated by PCR amplification of genomic DNA isolated from purified *C. trachomatis* serovars L2 and D, *C. pneumoniae*, *C. muridarum* and *C. caviae* with species specific primers (Table S1) and biotinylated using a BrightStar Psoralen-Biotin Nonisotopic Labeling Kit Ambion, Inc.). Probed membranes were washed and IhtA detected with the BrightStar BioDetect Nonisotopic detection kit (Ambion, Inc.).

### Clones

The plasmids pTet, pLac, serovar L2 *hctA*pTet and *ihtA*pLac have been described elsewhere [16], [17]. To clone the different species *hctA*, PCR fragments from *C. trachomatis* serovar D, *C. pneumoniae*, *C. muridarum* and *C. caviae* genomic DNA were generated using the primers indicated in Table S1. *hctA* fragments from all species except *C. pneumoniae* were cloned into the *Kpn*1/*Pst*1 sites of pTet. *C. pneumoniae hctA* was cloned into the *Kpn*1/*Not*1 sites. To generate *ihtA* clones from each species, *ihtA* and 5′UTR was PCR amplified using the primers indicated in Table S1 and ligated into the *Kpn*1/*Pst*1 sites of pLac.

### *E. coli* Rescue Conditions

*E. coli* rescue assays were performed as previously described with a few modifications [16], [17]. DH5aPRO *E. coli* (Clontech) cultures co-expressing the appropriate *hctA* and *ihtA* constructs were grown in triplicate overnight at 37°C in Luria–Bertani (LB) containing 100 µg/ml carbenicillin (cb), 34 µg/ml chloramphenicol (cm) and 50 µg/ml spectinomycin (spec). Cultures were then diluted 1∶2000, split into two tubes and one half induced to express HctA with 100 ng/ml anhydrotetracycline (aTc) and incubated with shaking at 30°C for 16 h. There is no need to induce IhtA as expression is constitutive. Growth was determined spectrophotometrically at OD_550_ and the ability of a particular construct to rescue the lethal phenotype of HctA was expressed as a percentage of the ratio between the induced and uninduced samples.

### Staining

*E. coli* from rescue experiments were pelleted and washed twice in 1 ml PBS. Pellets were resuspended in 4% paraformaldehyde and incubated at RT for 20 min. Samples were washed twice in PBS prior to incubation with 1∶500 dilution of DRAQ5 (Biostatus) for 30 min. Samples were again pelleted, washed in PBS, resuspended in Mowiol 4–88 (Calbiochem) and mounted on glass slides. Images were acquired using a spinning disk confocal system connected to a Leica DMIRB microscope, equipped with a Photometrics cascade-cooled EMCCD camera, under the control of the Open Source software package µManager (http://www.micro- manager.org/). Images were processed using the image analysis software ImageJ (http://rsb.info.nih.gov/ij/).

### Biolayer Interferometry

Sense IhtA and *hctA* transcripts were synthesized from the T7 promoter of PCR amplified fragments generated from serovars L2 and D, *C. pneumoniae*, *C. muridarum* and *C. caviae* genomic DNA using the primers described in Table S1. Antisense IhtA (scrambled control) was synthesized from from the T7 promoter of a PCR amplified fragment generated from serovar L2. The *hctA* transcripts were designed to include 5′ UTR starting at the transcription start site (TSS) [37] and an addition 21 nt “A” tail used to bind the transcript to the streptavidin biosensor tips. Run off transcripts were prepared using the MEGAshortscript T7 kit as described by the manufacturer (Ambion Inc.).

Biolayer interferometry studies of RNA:RNA interactions were performed using the Octet QKe (ForteBio, Menlo Park, CA). To anneal the ligand (*hctA* message) to the streptavidin biosensor tips, 150 nM *hctA* transcript, 150 nM 5′ biotinylated oligo T (complimentary to the 3′ “A” tail), 1xRNA Binding Buffer (RBB, 10 mM Tris-HCl pH 8, 125 nM NaCl, 125 mM KCl, 25 mM MgCl2) were combined, heated for 1 min at 90°C and allowed to cool slowly. During this time, SA biosensor tips were equilibrated in RBB buffer for 15 min. RNA annealed to biotinylated oligo was loaded onto the SA tips for 15 min or until saturation. RNA loaded tips were then soaked in RBB buffer for 5 min prior to incubation with 1500 nM IhtA which had been heated at 90°C for 1 min and allowed to cool to RT. The change in internally reflected light attributable to RNA:RNA interactions was collected in real time for 20 minutes using the software provided with the Octet QKe.

## Supporting Information

Table S1 Primers for cloning and in vitro transcription.
(PDF)
