# Supplementary material for: Translation Inhibition of the Developmental Cycle Protein HctA by the Small RNA IhtA Is Conserved across Chlamydia
Source: PLoS One. 2012 Oct 11;7(10):e47439. doi: 10.1371/journal.pone.0047439 (PMC3469542; doi:10.1371/journal.pone.0047439)
Supplement: Table S1 — Primers for cloning and in vitro transcription. (PDF) [file pone.0047439.s001.pdf]

Table S1. Primers for cloning and Invitro transcription

Primer	Sequence
hctA forward (common)	ATGGCGCTAAAAGATACGGC
D and L2 *hctA* reverse	TTATTTTTTTGTTGAGCGAGTTTTTTTCGCTG
muridarum *hctA* reverse	TTATTTTTTTGTTGAACGAACTTTTTTCG
caviae *hctA* reverse	TTATTTTCTTAGGGAGCGAGAAGAATTTTTTTTTGG
pneumoniae *hctA* reverse	TTATTTTCTAAATCCGCGTGCTTTGGAAGG
D *ihtA* forward	TATTCGTGATAAACTGACAAAAATTGTATCC
D *ihtA* reverse	CCTACGCGTTACGAATGCGTTGCTCTACC
caviae *ihtA* forward	TTTACAAGTTCCAGATGGATGTTTCTTAG
caviae *ihtA* reverse	CTTTAGAAGTCCCTGCCTTTCCACTAAGAAG
muridarum *ihtA* forward	TAATCAAAGGACTCTTTAGCCGAACAATCG
muridarum *ihtA* reverse	ACTGAGCTATTTAGGCTAACTTACAAAAAGC
pneumoniae *ihtA* forward	ACGCAATATCATGATGTTTATTAAGCCTAAGG
pneumoniae *ihtA* reverse	CCAACTGAGCTATTTAGGCATGTCGTAGG
**Invitro transcription**	
T7 L2, D forward	TAATACGACTCACTATAGGGAAGTTGGTATTCTAACGCCATGGAATAGC
T7 muridarum *ihtA* forward	TAATACGACTCACTATAGGGATAAAGGTTGCAAGTTGGTATTCTAACGCCATGG
T7 caviae *ihtA* forward	TAATACGACTCACTATAGGGATAGAAAAAATAAGATGATATTCTACGCCATGG
T7 pneumoniae i*htA* forward	TAATACGACTCACTATAGGGATAAAAGAGGAAGATGATATTCTCCGCCGTGG
T7 *ihtA* antisense forward	TAATACGACTCACTATAGGGCCATAAAAAGCCAAGAGAACCGGAGATACG
L2, D *ihtA* reverse	AAAGCCAAGAGAACCGGAGATACGGCTAACTCC
muridarum *ihtA* reverse	AAAAAGCCAAGAGAACTGGAGACACGGCTAAC
caviae *ihtA* reverse	AAAGCCAAGAAAACAACAATCATACG
pneumoniae *ihtA* reverse	AAAGCCAAGAAAACATAAAGTTATAC
IhtA sense reverse	AAAGCCAAGAGAACCGGAGATACG
IhtA antisense reverse	AAGTTGGTATTCTAACGCCATGGAATAGC
T7 L2, D *hctA* forward	TAATACGACTCACTATAGGGTTAAAACTGAAAAAAATAGTTTAAAACAACAAC
T7 muridarum *hctA* forward	TAATACGACTCACTATAGGGTTAAAACTAAAAAAAATAGTTTAAAACAACG
T7 caviae *hctA* forward	TAATACGACTCACTATAGGGTTAAAAATTAAAACAAGTAAAAATAGTTTAAAAC
T7 pneumoniae *hctA* forward	TAATACGACTCACTATAGGGATTAAAACAAGTAAAAATAGTTTAAAACAGC
L2,D *hctA* reverse	TTTTTTTTTTTTTTTTTTTTTTTATTTTTTTGTTGAGCGAGTTTTTTTCGCTG
muridarum *hctA* reverse	TTTTTTTTTTTTTTTTTTTTTTTATTTTTTTGTTGAACGAACTTTTTTCGTCACTTTAGC
caviae *hctA* reverse	TTTTTTTTTTTTTTTTTTTTTTTATTTTCTAAATCCGCGTGCTTTGGAAGG
pneumoniae *hctA* reverse	TTTTTTTTTTTTTTTTTTTTTTTATTTTCTAAATCCGCGTGCTTTGGAAGG

Underlined sequence indicates the T7 promoter.
